# Numerous Paragraphs of Interest

**Published:** 1895-09

**Authors:** 


### Medical News and Miscellany.

**Physicians** who contemplate taking a post-graduate course this fall, can learn something to their advantage by dropping a card to the managing editor of the JOURNAL, stating about when they expect to go.

**Horse Meat Test.**—The *Medical Record's* correspondent from Paris says that horse bouillon can be distinguished from that made of beef or mutton by means of iodine. The horse bouillon colors reaction paper red-brown, shading toward violet. This should be regarded as valuable information by those who object to eating horse flesh.

**Wheels in His Head.**—The *Lancet-Clinic* reports the case of a "human time-keeper;" a most remarkable case, aud nothing like it recorded. A negro man, aged 56, had, in boyhood, been struck on the head by a pick, resulting in a depressed and adherent cicicatrix. When asked the time—night or day—he would place his finger on this spot and answer, always giving the correct time, or within a few minutes of it- The writer, Dr. Culberson, of Zanesville, Ohio, tries to explain it by "unconscious cerebration." No such thing; he has wheels in there.

**Insurance.**—Our friend, Dr. R. Harvey Reed, of the *Columbus Medical Journal*, is reaping a harvest of a very doubtful kind of glory, the fruits of his advocating reform in the matter of vicarious and amateur medical referees for insurance companies; of instituting the practice of charging a fee for the particular kind of service involved as well as for other services, rendered insurance companies. The majority of the profession are with him, we think. Although the TEXAS MEDICAL JOURNAL was the first, we believe, to call attention to this kind of imposition on the doctors, and to advocate making a charge for the service, we are not sorry that the "credit" for it has been given to Dr. Reed; he is certainly welcome to all the glory there is in it.

---

**Dr. E. R. Walker,** of Cistern, Texas, we regret to hear, is down with a severe attack of typhoid fever; but latest advices report his condition—third week—as favorable, and strong hopes of his recovery are entertained.

---

**Those** who read this will readily understand for whom it is intended. Every subscriber knows if he is in arrears with the JOURNAL, at least, to any extent. To all who are, we make an earnest appeal to pay. We pay cash for each edition, and it is a heavy burden. Our subscribers have had the benefit of this and of our labors, some of them for *several years*, and we must call on them to help us cary the load. Kindly make an effort; make it a point, immediately upon reading this, to go down into your pants pocket, or wherever else you carry your surplus wealth, and divide with us. The JOURNAL will gratefully acknowledge remittances of any amount, whether in full or on account. Now, don't postpone it; attend to it at once and greatly oblige us.

---

**We** regret to learn that Dr. T. C. Karnes, the assistant superintendent Southwest Texas insane asylum, has been compelled to resign on account of the wound received last fall while hunting, an acccount of which appeared in the JOURNAL.

---

**What** funny names doctors do have. On our subscription list alone there are all the colors, nearly—all the points of the compas, and a few proverbs. We have Drs. "Bibb and Tucker;" Drs. North, East, West, and Southgate; Drs. Brown, Red, Gray, Black, White, Green, and Dunn. We did know a Mr. Blue—he blew his brains out;—Dr. Goforth and Dr. Cum-ins.

Then we have them in Cupples, Triplets and Single-ton; Drs. Barker and Bit-ting; Rolling, Stone, and Moss; Grubs and Fly; Some are Blunt and Dull; others are Sharp and Keen. There is

Field, Hunt and Gunn, Rod-ney and Bass; to say nothing of Young, Peeples and Small, Frey.

Old man Aiken wanted his nephew, Dr. Paine, as a condition to leaving him all his wealth, to take his name; hyphenate them thus: "Aiken-Paine," and the nephew told him as he was a doctor he saw so much of that sort of thing he was disgusted, and would see him d—rowned first; fact, aching pains galore in our calling, eh?

---

**Dr. B. D. Smith,** of St. Elmo, Texas, has gone to Boston, to attend the tri-ennial conclave of Knights Templar. From Boston he will go to New York and take a course at the Polyclinic.

---

**Removal.**—A. L. Hummel, the expert medical advertiser of Philadelphia, one of the most enterprising and successful men in the business, has found it necessary, in order to keep up with his increasing business, to remove to New York. On the 22d of August, ult., he opened up his office at 108 Fulton street.

---

**An Innovation—Excellent Facilities Now Offered by the Iron Mountain Route to Travelers En Route to the North and East.**—With the change of time on the Iron Mountain Route, effective June 30th, residents of Texas have been put in the closest possible communication with Memhhis, St. Louis, Chicago, Cincinnati, Philadelphia, Boston, New York and the Atlantic seaboard. The most noteworthy feature inaugurated is that of train No. 54. This is known as the "Texas-New York Flyer," carrying as it does, through Pullman buffet sleeping cars from El Paso, Fort Worth, Dallas, and way points to Little Rock and St. Louis, where direct connections are made with the fast Vandalia train for New York, and midnight special over the Chicago & Alton R. R. for Chicago.

Leave Texarkana 4:10 a. m., arrive at St. Louis 10:05 p. m., arrive at Chicago 8:00 a. m. next morning, New York 7:43 the second morning out. A double daily line of through Pullman buffet sleeping cars is also operated between Galveston and St. Louis, via International & Great Northern R. R., one line, and Gulf, Colorado & Sante Fe, and Houston & Texas Central the other line. Day coaches are also run between Fort Worth, Dallas, Texarkana and Memphis, arriving at the Mississippi River Gate-way at 4:30 p. m. This in addition to the old established service between Laredo and San Antonio, gives Texas the best outlet to the North it has ever known.

Mr. John C. Lewis, traveling passenger agent of the Iron Mountain Route, Austin, Texas, seems very enthusiastic over the new card, and reports a most encouraging travel, particularly on No. 54, the fast "Texas-New York Flyer," which has been so extensively advertised.

---

**Contributions** to our pages are solicited. Every doctor meets in practice with something, a knowledge of which would be interesting and instructive to others. Let him report it through the JOURNAL. Never mind if you are not an experienced writer. Jot it down as you would tell it to a friend; send to us, and we will put it in shape.

---

**Data** have been obtained from the following named States: Alabama, Minnesota, Maryland, North Dakota, North Carolina, New York, New Jersey, Virginia and Washington.

The subjoined table indicates briefly the work of these boards:

| State. | Examined. | Licensed. | Rejected. | Per cent. |
|---|---|---|---|---|
| Alabama | 647 | 558 | 89 | 0.862 |
| Maryland | 150 | 105 | 25 | 0.806 |
| Minnesota | 641 | 499 | 142 | 0.778 |
| New York | 967 | 797 | 170 | 0.824 |
| New Jersey | 447 | 417 | 30 | 0.955 |
| North Carolina | 615 | 508 | 207 | 0.71 |
| North Dakota | 81 | 76 | 5 | 0.938 |
| Virginia | 835 | 613 | 222 | 0.734 |
| Washington | 207 | 167 | 40 | 0.806 |
| Totals | 4670 | 3640 | 930 | 0.822 |

It will be observed that of four thousand six hundred and seventy persons examined but eighty-two and two-tenths per cent were successful in securing a license. The nine hundred and thirty unsuccessful applicants have, we doubt not, principally located in States not protected by this form of legislation.

[No doubt a large part of this class came to Texas. We have no law here to hinder. –ED.]

---

**A Doctor Whipped.**—A telegram to the *Statesman*, of August 26, states that a Miss Nanie Benson who had formerly worked in the office of Dr. L. M. Bridgers, of Brenham, met that gentleman in the street, and with a six-shooter in one hand and a wagon whip in the other, proceeded to give him a sound whipping. While the proprietor of the hotel, a Mr. Manglier (ought to have been *Mangler*), used a cane on the doctor, the girl laid on the whip. Cause said to be letters the doctor wrote her after she had quit. The doctor never had a better chance in his life to run than was here presented; and a good run is better than a bad stand. That's the kind of girls we have in Texas, a terror to "mashers."

---

**Dr. E. M. Albers** has removed from Victoria to William Penn, Washington county, Texas.

---

**Proportion of Physicians to Population:**

| | |
|---|---|
| Austio-Hungarian Empire | 1 to 3 857 |
| Belgium | 1 to 2,841 |
| France | 1 to 2,666 |
| German Empire | 1 to 3,038 |
| Italy | 1 to 3,536 |
| Netherlands | 1 to 2,484 |
| Norway | 1 to 3,961 |
| Russia | 1 to 8,551 |
| Spain | 1 to 3,375 |
| United States | 1 to 500 |

---

**Camp Jenner.**—The camp of the returning negro colonists, near Eagle Pass, now in the hands of the M. H. S., has been named "Camp Jenner," on account of the large amount of vaccination that has been necessary, we suppose. To date, August 26, the number of arrivals of these negroes from Mexico is 396. Of these 166 have had small-pox, with 45 deaths to date. Not a case has occurred in the State outside the camp; it is completely jugulated; it can't get out.

